# Association of novel lipid markers with cardiovascular and cerebrovascular disease risk: A cross-sectional NHANES 1999 to 2018 study

**DOI:** 10.1097/MD.0000000000045025

**Published:** 2026-02-28

**Authors:** Limei Guan, Tonglu Su, Shilong Xin, Yu Zhang, Benyin Wang, Xiaojuan Wang, Yingli Zhang, Tao Sun

**Affiliations:** a Department of Geriatrics, First Affiliated Hospital of Heilongjiang University of Chinese Medicine, Harbin, China; b Heilongjiang University of Chinese Medicine, Harbin, China; c Department of Cardiology, Harbin Red Cross Central Hospital, Harbin, China; d Department of Orthopedics II, Xiangfang District People’s Hospital, Harbin, China.

**Keywords:** atherogenic index of plasma, cardiovascular and cerebrovascular diseases, dyslipidemia, lipid accumulation product, non-HDL-C/HDL-C ratio

## Abstract

Dyslipidemia is central to cardiovascular and cerebrovascular diseases (CCVD). This study investigates associations of novel lipid markers – NHHR (non-HDL-C/HDL-C ratio), atherogenic index of plasma (AIP), and lipid accumulation product (LAP) – with CCVD risks using National Health and Nutrition Examination Survey data, comparing their predictive performance with traditional lipid and obesity indices. Data from 18,996 adults were analyzed. Multivariable logistic regression evaluated associations of NHHR/AIP/LAP with hypertension, heart failure, coronary heart disease, angina, and stroke. Restricted cubic splines (RCSs) tested nonlinearity, receiver operating characteristic curves assessed predictive ability, and subgroup analyses examined heterogeneity. Fully adjusted models revealed significant associations of NHHR and AIP with hypertension, heart failure, coronary heart disease, angina, and stroke, while LAP showed no link to stroke. RCS indicated nonlinear NHHR-hypertension and NHHR-coronary heart disease relationships (*P* for nonlinear < .05). AIP exhibited nonlinear associations with hypertension and stroke, while LAP demonstrated threshold effects for hypertension, coronary heart disease, and angina. Subgroup analyses identified interactions: sex and diabetes modified NHHR-hypertension associations (*P* < .05); AIP-hypertension links varied by sex, education, smoking, and body mass index (BMI); LAP-hypertension associations differed by sex and BMI (*P* < .05). NHHR, AIP, and LAP independently predict CCVD risks, with AIP showing optimal stability, surpassing traditional markers. Prospective studies are needed to confirm causality.

## 1. Introduction

Cardiovascular and cerebrovascular diseases (CCVDs) represent the leading cause of chronic disease mortality worldwide. According to the World Health Organization and the Global Burden of Disease Study, CCVDs accounted for approximately 33% of global deaths, surpassing mortality rates from malignancies and respiratory diseases.^[1]^ Epidemiological data indicate 11.4 million new coronary heart disease cases and 13.7 million new stroke cases annually, underscoring the critical public health burden.^[2]^ The high morbidity, suboptimal disease control, and extensive societal impact of CCVDs have precipitated severe economic consequences, with direct medical costs exceeding $430 billion and indirect losses reaching $1.3 trillion annually in the United States alone.^[3]^ Accelerated by lifestyle modifications and population aging, the escalating economic burden associated with CCVD treatment and post-disease rehabilitation necessitates urgent emphasis on early diagnosis and preventive interventions.

Abnormal lipid metabolism constitutes a central pathophysiological mechanism underlying CCVD development.^[4,5]^ Clinical evidence identifies dysregulated lipoprotein profiles as critical risk biomarkers for CCVD, particularly in high-risk populations predisposed to lethal complications.^[6,7]^ Atherogenesis is initiated by elevated low-density lipoprotein cholesterol (LDL-C) levels, wherein LDL-C particles infiltrate the subendothelial space and undergo oxidative modification to form oxidized LDL.^[8]^ oxidized LDL drives early plaque progression through multiple mechanisms: inducing monocyte-to-macrophage differentiation, facilitating foam cell formation via lipid phagocytosis, and activating pro-inflammatory pathways such as nuclear factor κ-light-chain-enhancer of activated B cells to amplify localized inflammatory responses and lipid core accumulation.^[9,10]^ Concurrently, hypertriglyceridemia exacerbates vascular pathology by increasing triglyceride-rich lipoprotein (TRL) production, which promotes endothelial dysfunction and lipid infiltration into arterial walls.^[11]^ A clinical study demonstrated a 1.7-fold elevation in ischemic heart disease risk per 1 mmol/L increment in triglycerides (TG).^[12]^ Compounding these effects, high-density lipoprotein cholesterol (HDL-C) dysfunction – manifested as impaired cholesterol reverse transport capacity – leads to peripheral cholesterol deposition, while diminished anti-inflammatory, antioxidant, and endothelial-protective properties fail to counterbalance the pro-atherogenic effects of LDL-C and TRLs.^[13]^ Prospective cohort studies further validate the independent predictive value of abnormal total cholesterol (TC)-to-HDL-C ratios for cardiovascular events.^[14]^ The synergistic interplay between LDL-C/TG-driven atherogenesis and HDL-C functional deficiency establishes the cornerstone of CCVD pathogenesis.

Emerging lipid biomarkers – non-HDL-C to HDL-C ratio (NHHR), atherogenic index of plasma (AIP), and lipid accumulation product (LAP) – integrate conventional lipid parameters (TC, TG, HDL-C) with anthropometric indices (WC) to refine metabolic risk stratification. Compared to traditional diagnostic approaches, these composite indices demonstrate enhanced accessibility and predictive utility across diverse conditions, including depression, diabetic nephropathy, metabolic syndrome, sarcopenia, and cardiovascular diseases.^[15–19]^

Leveraging data from the National Health and Nutrition Examination Survey (NHANES), this study investigates the correlation between these novel lipid markers (NHHR, AIP, LAP) and CCVD outcomes. Our analysis evaluates the clinical feasibility of these indices for monitoring and predicting CCVD risk, aiming to establish simplified assessment tools for early risk stratification and targeted intervention in CCVD management.

## 2. Methods

### 2.1. Study population

**Figure 1.**
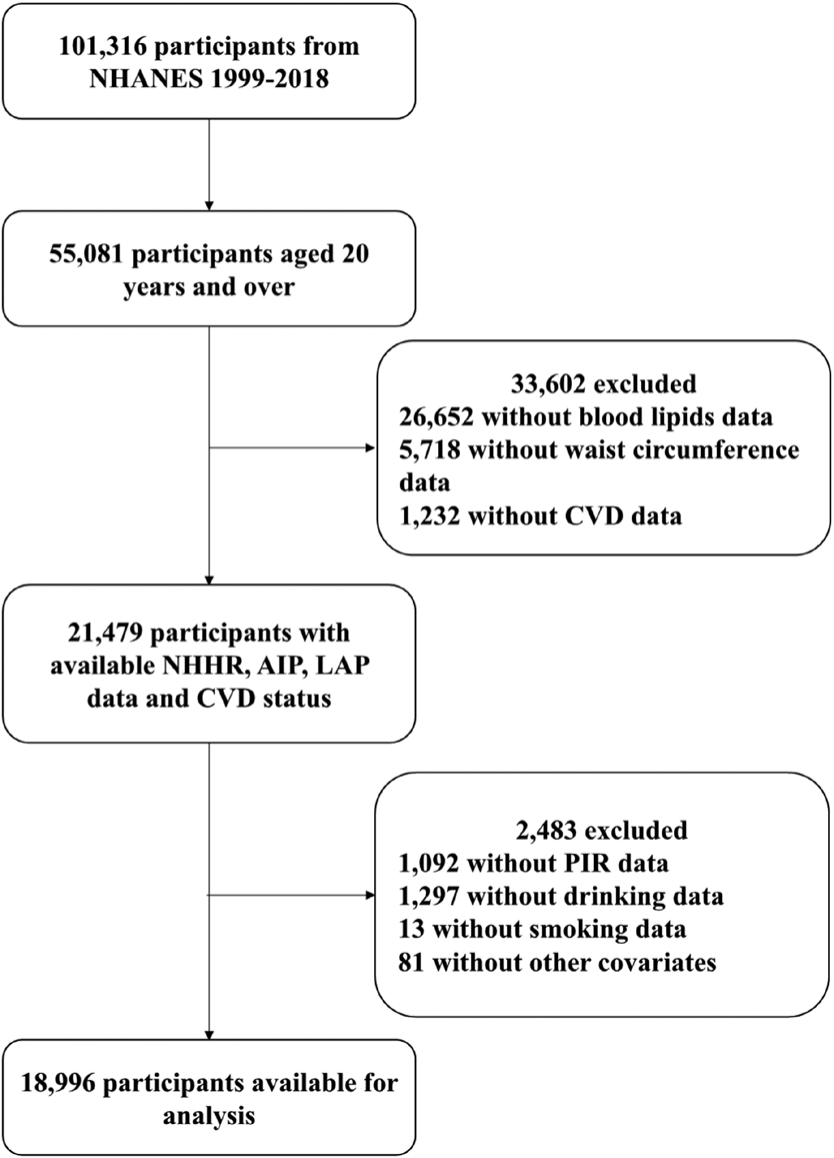
Diagram of participant enrollment process.

All participant data were extracted from the NHANES database spanning 1999 to 2018 (detailed data accessible at https://www.cdc.gov/nchs/nhanes/index.htm). This study involving human participants were reviewed and approved by the Research Ethics Review Board of the NCHS, and all participants provided written informed consent during the survey. Eligible participants included in this study had complete demographic records, standardized anthropometric measurements, blood pressure readings, lipid profiles, fasting glucose and insulin levels, and medical history documentation. Exclusion criteria were: age < 20 years; missing blood pressure data or self-reported diagnoses of heart failure, hypertension, coronary heart disease, angina, or stroke; incomplete data for calculating NHHR, AIP, or LAP; and absence of covariate data, including poverty-income ratio (PIR), marital status, educational attainment, alcohol consumption, smoking history, body mass index (BMI), and diabetes status. A flowchart detailing the participant selection process is provided in Figure 1.

### 2.2. Exposure variables: NHHR, AIP, and LAP

The novel lipid markers – non-HDL-C to HDL-C ratio (NHHR), AIP, and LAP – were calculated using laboratory and anthropometric data from NHANES, including HDL-C, TG, TC, and waist circumference (WC). The computational formulae are as follows:

**NHHR**: [TC (mg/dL)-HDL-C (mg/dL)]/HDL-C (mg/dL)^[20]^。

**AIP**: log10[TG (mmol/L)/HDL-C (mmol/L)]^[21]^。

**LAP**: Male = (WC-65)* TG (mmol/L); Female = (WC-58)* TG (mmol/L)^[22]^。

### 2.3. Outcome variables: hypertension, heart failure, coronary heart disease, angina, and stroke

Hypertension was defined as: physician-diagnosed hypertension; current use of antihypertensive medications; or average systolic blood pressure ≥ 140 mm Hg or diastolic blood pressure ≥ 90 mm Hg across 3 standardized measurements. Blood pressure measurements were obtained by trained examiners following a strict protocol: participants rested for ≥5 minutes prior to measurement, with 3 consecutive readings recorded. Diagnoses of heart failure, coronary heart disease, angina, and stroke were derived from self-reported responses to the question: “Has a doctor or healthcare professional ever told you that you have [condition]?” Affirmative responses classified participants as cases for each respective outcome.

### 2.4. Covariates

Potential confounders influencing metabolic indices and cardiovascular disease (CVD) associations were adjusted, including: Sociodemographics: Age (years), sex (male/female), race/ethnicity (Mexican American, Non-Hispanic White, Non-Hispanic Black, Other Hispanic, Other Race), marital status (married/unmarried), education level (<9th grade, 9–11th grade, high school graduate, some college, college graduate), and PIR. Lifestyle factors: Smoking status (never: <100 lifetime cigarettes; ever: ≥100 cigarettes) and alcohol consumption (nondrinker: <12 drinks/year; drinker: ≥12 drinks/year). Clinical measures: BMI (kg/m^2^), WC (cm), blood pressure, fasting glucose (mmol/L), TG (mmol/L), and HDL-C (mmol/L). Anthropometric and biochemical measurements were obtained by certified technicians at mobile examination centers, with blood samples processed at the University of Minnesota. Diabetes mellitus was defined as: fasting glucose ≥ 7.0 mmol/L; insulin use; oral hypoglycemic therapy; or self-reported diagnosis.

### 2.5. Statistical analysis

All analyses incorporated NHANES sampling weights. WC was categorized into quartiles (Q1–Q4) due to its strong association with dyslipidemia. Continuous variables were expressed as mean ± standard deviation, and categorical variables as percentages. Weighted linear regression and χ^2^ tests compared baseline characteristics across WC quartiles. Multivariable logistic regression models evaluated associations between NHHR/AIP/LAP and each CVD outcome. Model 1: Unadjusted; Model 2: Adjusted for sex, age, race; Model 3: Fully adjusted for sex, age, race, education, PIR, marital status, BMI, smoking, alcohol use, and diabetes. Nonlinear relationships were assessed using restricted cubic splines (RCSs) and threshold effect analyses. Subgroup analyses stratified by sex, age, race, education, marital status, PIR, smoking, alcohol use, and diabetes evaluated effect heterogeneity via interaction terms. Statistical significance was set at *P* < .05. Analyses were performed using R v4.2.1 and EmpowerStats v2.0.

## 3. Results

### 3.1. Baseline characteristics

A total of 18,996 adults were included in this study based on the inclusion and exclusion criteria. The results showed that the mean age of participants was 49.40 ± 17.93 years, with 9360 males (49.27%) and 9636 females (50.73%). The cohort comprised 3305 Mexican Americans (17.40%), 1598 individuals of other races (8.41%), 8928 non-Hispanic Whites (47.00%), 3676 non-Hispanic Blacks (19.35%), and 1489 other Hispanics (7.84%). The mean BMI of the population was 28.90 ± 6.62 kg/m^2^ (Table 1).

**Table 1:** Weighted comparison in basic characteristics.

Variables	Total (n = 18,996)	Q1 < 87.6 (n = 4721)	Q2 87.7–97.7 (n = 4772)	Q3 97.8–108.4 (n = 4741)	Q4 >108.4 (n = 4762)	*P*-value
Age (yr)	49.40 ± 17.93	42.63 ± 18.06	49.68 ± 17.68	52.87 ± 17.40	52.39 ± 16.69	<.001
PIR	2.58 ± 1.62	2.64 ± 1.67	2.62 ± 1.62	2.58 ± 1.60	2.50 ± 1.59	<.001
BMI (kg/m^2^)	28.90 ± 6.62	22.36 ± 2.53	26.43 ± 2.62	29.79 ± 3.10	36.96 ± 6.16	<.001
TG (mmol/L)	1.49 ± 1.14	1.09 ± 0.66	1.49 ± 1.13	1.64 ± 1.27	1.74 ± 1.51	<.001
LAP	6.06 ± 6.63	2.11 ± 1.63	4.65 ± 3.78	7.04 ± 6.26	10.41 ± 9.07	<.001
HDL-C (mg/dL)	53.65 ± 16.06	61.82 ± 17.02	54.54 ± 15.90	50.69 ± 14.52	47.61 ± 12.92	<.001
TC (mg/dL)	195.49 ± 42.61	188.18 ± 39.33	199.52 ± 43.43	200.36 ± 43.04	193.84 ± 43.35	<.001
NHHR	2.92 ± 1.40	2.23 ± 1.03	2.91 ± 1.29	3.23 ± 1.49	3.32 ± 1.47	<.001
AIP	0.33 ± 0.34	0.15 ± 0.29	0.33 ± 0.32	0.42 ± 0.33	0.45 ± 0.32	<.001
LDL (mg/dL)	115.59 ± 35.75	111.75 ± 34.80	116.49 ± 35.86	117.14 ± 36.11	117.05 ± 35.97	<.001
SBP (mm Hg)	124.06 ± 19.12	122.76 ± 19.33	123.95 ± 18.84	124.80 ± 18.97	124.72 ± 19.27	<.001
DBP (mm Hg)	69.63 ± 13.21	69.05 ± 13.00	69.59 ± 13.27	69.84 ± 13.45	70.02 ± 13.10	.003
Fasting glucose (mmol/L)	5.99 ± 1.99	5.80 ± 1.66	5.97 ± 1.90	6.12 ± 2.21	6.07 ± 2.11	<.001
White blood cell count (1000 cells/µL)	6.87 ± 2.42	6.66 ± 2.73	6.85 ± 2.06	6.96 ± 2.11	7.00 ± 2.70	<.001
Lymphocyte percent (%)	2.03 ± 1.28	2.00 ± 1.90	2.02 ± 0.80	2.03 ± 0.84	2.07 ± 1.25	.041
Segmented neutrophils percent (%)	4.05 ± 1.75	3.89 ± 1.63	4.04 ± 1.63	4.13 ± 1.68	4.13 ± 2.02	<.001
Red blood cell count (million cells/µL)	4.71 ± 0.51	4.68 ± 0.51	4.72 ± 0.52	4.72 ± 0.52	4.72 ± 0.52	<.001
Platelet (1000 cells/µL)	249.43 ± 67.42	246.15 ± 66.25	250.13 ± 67.85	250.44 ± 67.90	251.00 ± 67.59	.002
Sex, n (%)						
Male	9360 (49.27)	1756 (37.20)	2400 (50.29)	2642 (55.73)	2562 (53.80)	<.001
Female	9636 (50.73)	2965 (62.80)	2372 (49.71)	2099 (44.27)	2200 (46.20)	
Race, n (%)						
Mexican American	3305 (17.40)	634 (13.43)	943 (19.76)	994 (20.97)	734 (15.41)	<.001
Other Hispanic	1489 (7.84)	334 (7.07)	421 (8.82)	399 (8.42)	335 (7.03)	
Non-Hispanic White	8928 (47.00)	2147 (45.48)	2119 (44.40)	2232 (47.08)	2430 (51.03)	
Non-Hispanic Black	3676 (19.35)	914 (19.36)	821 (17.20)	866 (18.27)	1075 (22.57)	
Other race	1598 (8.41)	692 (14.66)	468 (9.81)	250 (5.27)	188 (3.95)	
Level of education, n (%)						
<9th	2053 (10.81)	362 (7.67)	564 (11.82)	651 (13.73)	476 (10.00)	<.001
9–11th	2783 (14.65)	625 (13.24)	718 (15.05)	686 (14.47)	754 (15.83)	
High school	4386 (23.09)	1031 (21.84)	1053 (22.07)	1151 (24.28)	1151 (24.17)	
Some college	5490 (28.90)	1373 (29.08)	1302 (27.28)	1316 (27.76)	1499 (31.48)	
College graduate	4284 (22.55)	1330 (28.17)	1135 (23.78)	937 (19.76)	882 (18.52)	
Marry, n (%)						
Married	10,280 (54.12)	2157 (45.69)	2675 (56.06)	2811 (59.29)	2637 (55.38)	<.001
Unmarried	8716 (45.88)	2564 (54.31)	2097 (43.94)	1930 (40.71)	2125 (44.62)	
Hypertension, n (%)						
Yes	7889 (41.53)	1776 (37.62)	2003 (41.97)	2056 (43.37)	2054 (43.13)	<.001
No	11,107 (58.47)	2945 (62.38)	2769 (58.03)	2685 (56.63)	2708 (56.87)	
Drinking, n (%)						
Yes	13,901 (73.18)	3503 (74.20)	3538 (74.14)	3446 (72.69)	3414 (71.69)	.013
No	5095 (26.82)	1218 (25.80)	1234 (25.86)	1295 (27.31)	1348 (28.31)	
Heart failure, n (%)						
Yes	564 (2.97)	139 (2.94)	120 (2.51)	154 (3.25)	151 (3.17)	.146
No	18,432 (97.03)	4582 (97.06)	4652 (97.49)	4587 (96.75)	4611 (96.83)	
Coronary heart disease, n (%)						
Yes	777 (4.09)	170 (3.60)	185 (3.88)	206 (4.35)	216 (4.54)	.085
No	18,219 (95.91)	4551 (96.40)	4587 (96.12)	4535 (95.65)	4546 (95.46)	
Angina, n (%)						
Yes	523 (2.75)	115 (2.44)	118 (2.47)	140 (2.95)	150 (3.15)	.083
No	18,473 (97.25)	4606 (97.56)	4654 (97.53)	4601 (97.05)	4612 (96.85)	
Stroke, n (%)						
Yes	669 (3.52)	149 (3.16)	141 (2.95)	176 (3.71)	203 (4.26)	.002
No	18,327 (96.48)	4572 (96.84)	4631 (97.05)	4565 (96.29)	4559 (95.74)	
Smoking, n (%)						
Yes	8872 (46.70)	2064 (43.72)	2231 (46.75)	2296 (48.43)	2281 (47.90)	<.001
No	10,124 (53.30)	2657 (56.28)	2541 (53.25)	2445 (51.57)	2481 (52.10)	
Diabetes, n (%)						
Yes	5226 (27.51)	1098 (23.26)	1314 (27.54)	1415 (29.85)	1399 (29.38)	<.001
No	13,770 (72.49)	3623 (76.74)	3458 (72.46)	3326 (70.15)	3363 (70.62)	

AIP = atherogenic index of plasma, BMI = body mass index, DBP = diastolic blood pressure, HDL-C = high-density lipoprotein cholesterol, LAP = lipid accumulation product, LDL = low-density lipoprotein, NHHR = non-HDL-C/HDL-C ratio, PIR = poverty-income ratio, SBP = systolic blood pressure, TC = total cholesterol, TG = triglycerides.

All laboratory and clinical indicators of participants are presented in Table 1. The population was categorized into 4 quartiles (Q1–Q4) based on WC: Q1 < 87.6 cm; Q2: 87.7–97.7 cm; Q3: 97.8–108.4 cm; Q4 > 108.4 cm. Demographic and clinical characteristics varied with increasing WC. Compared to participants with lower WC (Q1), those with higher WC (Q2–Q4) were older (*P* < .05), more likely to be male, elderly, non-Hispanic Black or White, alcohol consumers, and smokers (*P* < .05). Notably, individuals with higher WC exhibited elevated BMI, TG, fasting glucose levels, and reduced HDL-C levels compared to those with lower WC (*P* < .05), suggesting a higher likelihood of dyslipidemia in this group. Additionally, participants with higher WC had significantly greater prevalence of diabetes and stroke (*P* < .05) (Table 1).

### 3.2. Associations of NHHR, AIP, and LAP with cardiovascular and cerebrovascular diseases

The results suggested positive associations between lipid metabolism indices (NHHR, AIP, LAP) and hypertension, heart failure, coronary heart disease, and angina, with the exception of LAP for stroke (Tables 2–6). When analyzed as continuous variables in the fully adjusted Model 3 (sex, age, race, education, marital status, PIR, smoking, alcohol consumption, diabetes, and BMI), AIP showed significant positive correlations with hypertension, heart failure, coronary heart disease, angina, and stroke, while LAP and NHHR exhibited no significant association with stroke. However, logistic regression analyses for cardiovascular diseases (hypertension, heart failure, coronary heart disease, angina) revealed significant associations between NHHR/LAP and these outcomes. Specifically, each unit increase in NHHR, AIP, and LAP was associated with a 9% (OR = 1.09; 95% CI: 1.07–1.11, *P* < .001), 113% (OR = 2.13; 95% CI: 1.94–2.33, *P* < .001), and 14% (OR = 1.14; 95% CI: 1.08–1.20, *P* < .001) elevation in hypertension risk, respectively (Table 2). For heart failure, each unit increase corresponded to a 10% (OR = 1.10; 95% CI: 1.04–1.16, *P* = .001), 97% (OR = 1.97; 95% CI: 1.54–2.52, *P* < .001), and 12% (OR = 1.12; 95% CI: 1.03–1.22, *P* = .008) increase in risk (Table 3). Coronary heart disease risk rose by 5% (OR = 1.05; 95% CI: 1.00–1.10, *P* = .039), 84% (OR = 1.84; 95% CI: 1.49–2.28, *P* < .001), and 12% (OR = 1.12; 95% CI: 1.01–1.23, *P* = .030) per unit increment (Table 4). Angina risk increased by 6% (OR = 1.06; 95% CI: 1.00–1.13, *P* = .036), 99% (OR = 1.99; 95% CI: 1.54–2.57, *P* < .001), and 10% (OR = 1.10; 95% CI: 1.00–1.20, *P* = .046) (Table 5). stroke by 32% (OR = 1.32; 95% CI: 1.04–1.67, *P* = .02) and 47% (OR = 1.47; 95% CI: 1.18–1.84, *P* = .001), with no significant association observed for LAP (*P* > .05) (Table 6).

**Table 2:** Association between NHHR, AIP, LAP and hypertension.

Hypertension	Model 1	Model 2	Model 3
NHHR continuous	1.10 (1.08, 1.12) <0.001	1.10 (1.08, 1.12) <0.001	1.09 (1.07, 1.11) <0.001
NHHR quartile			
<1.94	1	1	1
1.95–2.67	1.15 (1.06, 1.25) <0.001	1.15 (1.06, 1.25) 0.001	1.14 (1.05, 1.24) 0.002
2.68–3.62	1.27 (1.17, 1.38) <0.001	1.26 (1.16, 1.37) <0.001	1.23 (1.13, 1.34) <0.001
>3.62	1.48 (1.36, 1.61) <0.001	1.47 (1.35, 1.60) <0.001	1.42 (1.30, 1.55) <0.001
AIP Continuous	2.24 (2.05, 2.44) <0.001	2.23 (2.04, 2.44) <0.001	2.13 (1.94, 2.33) <0.001
AIP quartile			
<0.10	1	1	1
0.10–0.32	1.44 (1.33, 1.57) <0.001	1.43 (1.31, 1.55) <0.001	1.41 (1.29, 1.53) <0.001
0.33–0.55	1.78 (1.64, 1.94) <0.001	1.77 (1.63, 1.93) <0.001	1.72 (1.58, 1.88) <0.001
>0.55	2.06 (1.90, 2.24) <0.001	2.05 (1.88, 2.23) <0.001	1.95 (1.79, 2.13) <0.001
LAP continuous	1.15 (1.09, 1.20) <0.001	1.13 (1.08, 1.19) <0.001	1.14 (1.08, 1.20) <0.001
LAP quartile			
<2.45	1	1	1
2.46–4.45	1.21 (1.11, 1.32) <0.001	1.20 (1.10, 1.30) <0.001	1.24 (1.14, 1.36) <0.001
4.46–7.66	1.29 (1.19, 1.40) <0.001	1.27 (1.16, 1.38) <0.001	1.33 (1.21, 1.46) <0.001
>7.66	1.42 (1.31, 1.54) <0.001	1.39 (1.28, 1.52) <0.001	1.53 (1.37, 1.70) <0.001

AIP = atherogenic index of plasma, LAP = lipid accumulation product, HDL-C = high-density lipoprotein cholesterol, NHHR = non-HDL-C/HDL-C ratio.

**Table 3:** Association between NHHR, AIP, LAP and heart failure.

Heart failure	Model 1	Model 2	Model 3
NHHR continuous	1.10 (1.05, 1.16) <0.001	1.11 (1.05, 1.16) <0.001	1.10 (1.04, 1.16) <0.001
NHHR quartile			
<1.94	1	1	1
1.95–2.67	1.10 (0.85, 1.41) 0.481	1.10 (0.85, 1.42) 0.461	1.08 (0.84, 1.40) 0.559
2.68–3.62	1.22 (0.95, 1.56) 0.116	1.23 (0.96, 1.58) 0.107	1.18 (0.91, 1.52) 0.208
>3.62	1.53 (1.21, 1.94) <0.001	1.54 (1.21, 1.96) <0.001	1.46 (1.14, 1.87) 0.003
AIP continuous	2.09 (1.65, 2.65) <0.001	2.12 (1.67, 2.70) <0.001	1.97 (1.54, 2.52) <0.001
AIP quartile			
<0.10	1	1	1
0.10–0.32	1.29 (0.99, 1.68) 0.06	1.30 (0.99, 1.69) 0.057	1.26 (0.96, 1.65) 0.092
0.33–0.55	1.51 (1.17, 1.95) 0.002	1.52 (1.17, 1.97) 0.0025	1.45 (1.11, 1.88) 0.006
>0.55	1.96 (1.53, 2.50) <0.001	1.98 (1.54, 2.54) <0.001	1.83 (1.42, 2.36) <0.001
LAP continuous	1.12 (1.03, 1.22) 0.008	1.12 (1.03, 1.22) 0.009	1.12 (1.03, 1.22) 0.008
LAP quartile			
<2.45	1	1	1
2.46–4.45	1.01 (0.79, 1.29) 0.9492	1.01 (0.79, 1.31) 0.9148	1.01 (0.79, 1.31) 0.9234
4.46–7.66	1.20 (0.95, 1.53) 0.1279	1.22 (0.95, 1.56) 0.1204	1.21 (0.94, 1.55) 0.1312
>7.66	1.28 (1.01, 1.62) 0.042	1.31 (1.02, 1.67) 0.032	1.31 (1.03, 1.68) 0.031

AIP = atherogenic index of plasma, LAP = lipid accumulation product, HDL-C = high-density lipoprotein cholesterol, NHHR = non-HDL-C/HDL-C ratio.

**Table 4:** Association between NHHR, AIP, LAP and coronary heart disease.

Coronary heart disease	Model 1	Model 2	Model 3
NHHR continuous	1.07 (1.03, 1.12) 0.002	1.07 (1.02, 1.12) 0.005	1.05 (1.00, 1.10) 0.039
NHHR quartile			
<1.94	1	1	1
1.95–2.67	1.09 (0.88, 1.35) 0.442	1.08 (0.87, 1.34) 0.491	1.06 (0.85, 1.32) 0.602
2.68–3.62	1.31 (1.06, 1.62) 0.011	1.29 (1.05, 1.59) 0.018	1.24 (1.00, 1.54) 0.051
>3.62	1.46 (1.19, 1.79) <0.001	1.43 (1.16, 1.76) <0.001	1.34 (1.08, 1.67) 0.007
AIP continuous	2.03 (1.66, 2.50) <0.001	2.01 (1.63, 2.47) <0.001	1.84 (1.49, 2.28) <0.001
AIP quartile			
**<0.10**	1	1	1
0.10–0.32	1.29 (1.03, 1.63) 0.03	1.27 (1.01, 1.61) 0.042	1.23 (0.97, 1.55) 0.087
0.33–0.55	1.66 (1.33, 2.07) <0.001	1.65 (1.32, 2.06) <0.001	1.56 (1.24, 1.95) <0.001
>0.55	2.08 (1.68, 2.57) <0.001	2.04 (1.65, 2.53) <0.001	1.88 (1.51, 2.35) <0.001
LAP continuous	1.09 (1.01, 1.18) 0.036	1.09 (1.00, 1.19) 0.041	1.12 (1.01, 1.23) 0.03
LAP quartile			
<2.45	1	1	1
2.46–4.45	1.22 (0.99, 1.52) 0.065	1.24 (1.00, 1.54) 0.052	1.31 (1.04, 1.64) 0.020
4.46–7.66	1.22 (0.99, 1.52) 0.065	1.26 (1.01, 1.56) 0.04	1.37 (1.07, 1.74) 0.012
>7.66	1.51 (1.23, 1.86) <0.001	1.52 (1.23, 1.88) <0.001	1.76 (1.35, 2.28) <0.001

AIP = atherogenic index of plasma, LAP = lipid accumulation product, HDL-C = high-density lipoprotein cholesterol, NHHR = non-HDL-C/HDL-C ratio.

**Table 5:** Association between NHHR, AIP, LAP and angina.

Angina	Model 1	Model 2	Model 3
NHHR continuous	1.08 (1.02, 1.14) 0.007	1.07 (1.01, 1.13) 0.013	1.06 (1.00, 1.13) 0.036
NHHR quartile			
<1.94	1	1	1
1.95–2.67	1.15 (0.89, 1.49) 0.291	1.14 (0.88, 1.48) 0.331	1.12 (0.86, 1.46) 0.394
2.68–3.62	1.24 (0.96, 1.60) 0.103	1.22 (0.94, 1.58) 0.132	1.19 (0.92, 1.55) 0.191
>3.62	1.48 (1.15, 1.89) 0.002	1.44 (1.12, 1.85) 0.004	1.40 (1.08, 1.81) 0.011
AIP continuous	2.24 (1.75, 2.86) <0.001	2.19 (1.71, 2.81) <0.001	1.99 (1.54, 2.57) <0.001
AIP quartile			
<0.10	1	1	1
0.10–0.32	1.18 (0.88, 1.57) 0.2702	1.17 (0.87, 1.56) 0.2974	1.10 (0.82, 1.48) 0.5067
0.33–0.55	1.75 (1.34, 2.28) <0.001	1.73 (1.32, 2.26) <0.001	1.62 (1.23, 2.12) 0.0005
>0.55	2.16 (1.67, 2.80) <0.001	2.12 (1.63, 2.75) <0.001	1.92 (1.47, 2.51) <0.001
LAP continuous	1.10 (1.01, 1.21) 0.0343	1.13 (1.02, 1.25) 0.0184	1.10 (1.00, 1.20) 0.0460
LAP quartile			
<2.45	1	1	1
2.46–4.45	1.04 (0.80, 1.35) 0.7895	1.04 (0.80, 1.35) 0.7802	1.03 (0.79, 1.34) 0.8352
4.46–7.66	1.12 (0.86, 1.45) 0.3946	1.10 (0.84, 1.44) 0.4759	1.10 (0.85, 1.43) 0.4532
>7.66	1.50 (1.17, 1.91) 0.0011	1.46 (1.14, 1.88) 0.0031	1.47 (1.15, 1.88) 0.0019

AIP = atherogenic index of plasma, LAP = lipid accumulation product, HDL-C = high-density lipoprotein cholesterol, NHHR = non-HDL-C/HDL-C ratio.

**Table 6:** Association between NHHR, AIP, LAP and stroke.

Stroke	Model 1	Model 2	Model 3
NHHR continuous	1.05 (1.00, 1.10) 0.075	1.05 (1.00, 1.10) 0.075	1.02 (0.96, 1.07) 0.551
NHHR quartile			
<1.94	1	1	1
1.95–2.67	1.37 (1.09, 1.72) 0.008	1.37 (1.09, 1.73) 0.007	1.30 (1.03, 1.64) 0.026
2.68–3.62	1.45 (1.15, 1.82) 0.001	1.46 (1.16, 1.84) 0.001	1.32 (1.05, 1.67) 0.02
>3.62	1.37 (1.09, 1.73) 0.007	1.38 (1.10, 1.74) 0.006	1.21 (0.95, 1.53) 0.124
AIP continuous	1.55 (1.24, 1.94) <0.001	1.56 (1.25, 1.96) <0.001	1.33 (1.06, 1.68) 0.0155
AIP quartile			
<0.10	1	1	1
0.10–0.32	1.67 (1.32, 2.12) <0.001	1.69 (1.33, 2.15) <0.001	1.58 (1.24, 2.02) <0.001
0.33–0.55	1.70 (1.34, 2.15) <0.001	1.71 (1.35, 2.18) <0.001	1.57 (1.23, 1.99) 0.0003
>0.55	1.69 (1.33, 2.14) <0.001	1.71 (1.34, 2.17) <0.001	1.47 (1.15, 1.88) 0.002
LAP continuous	1.07 (0.97, 1.17) 0.180	1.07 (0.97, 1.17) 0.171	0.98 (0.86, 1.12) 0.777
LAP quartile			
<2.45	1	1	1
2.46–4.45	0.80 (0.63, 1.01) 0.0601	0.82 (0.65, 1.04) 0.097	0.74 (0.58, 0.94) 0.015
4.46–7.66	1.11 (0.89, 1.37) 0.353	1.14 (0.92, 1.42) 0.24	0.93 (0.73, 1.19) 0.567
>7.66	1.15 (0.93, 1.42) 0.194	1.18 (0.95, 1.47) 0.142	0.90 (0.68, 1.19) 0.462

AIP = atherogenic index of plasma, LAP = lipid accumulation product, HDL-C = high-density lipoprotein cholesterol, NHHR = non-HDL-C/HDL-C ratio.

To reduce bias and validate stability, sensitivity analyses categorized NHHR, AIP, and LAP into quartiles. Intriguingly, NHHR exhibited a significant association with stroke when analyzed as a quartile variable, whereas LAP showed no association with stroke in either continuous or categorical models (Table 6). Compared to participants with lower NHHR, AIP, and LAP levels, those in the highest quartiles demonstrated increased risks: hypertension by 42% (OR = 1.42; 95% CI: 1.30–1.55, *P* < .001), 95% (OR = 1.95; 95% CI: 1.79–2.13, *P* < .001), and 53% (OR = 1.53; 95% CI: 1.37–1.70, *P* < .001) (Table 2); heart failure by 46% (OR = 1.46; 95% CI: 1.14–1.87, *P* = .003), 83% (OR = 1.83; 95% CI: 1.42–2.36, *P* < .001), and 31% (OR = 1.31; 95% CI: 1.03–1.68, *P* = .03) (Table 3); coronary heart disease by 34% (OR = 1.34; 95% CI: 1.08–1.67, *P* = .007), 88% (OR = 1.88; 95% CI: 1.51–2.35, *P* < .001), and 76% (OR = 1.76; 95% CI: 1.35–2.28, *P* < .001) (Table 4); angina by 40% (OR = 1.40; 95% CI: 1.19–1.76, *P* = .0003), 92% (OR = 1.92; 95% CI: 1.68–2.57, *P* < .001), and 47% (OR = 1.47; 95% CI: 1.23–1.86, *P* = .001) (Table 5); stroke by 32% (OR = 1.32; 95% CI: 1.04–1.67, *P* = .02) and 47% (OR = 1.47; 95% CI: 1.18–1.84, *P* = .001), with no significant association observed for LAP (*P* > .05) (Table 6).

### 3.3. RCS curve analysis and nonlinear threshold effects

**Figure 2.**
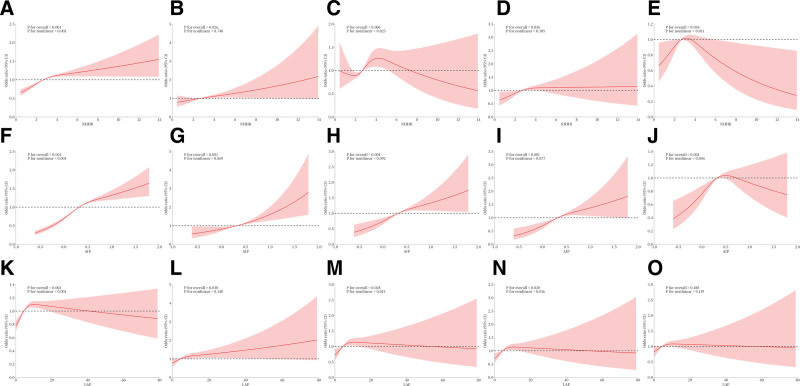
The association between NHHR, AIP, LAP and hypertension, heart failure, coronary heart disease, angina and stroke. (A) The association between NHHR and hypertension; (B) The association between NHHR and heart failure; (C) The association between NHHR and coronary heart disease; (D) The association between NHHR and angina; (E) The association between NHHR and stroke; (F) The association between AIP and hypertension; (G) The association between AIP and heart failure; (H) The association between AIP and coronary heart disease; (I) The association between AIP and angina; (J) The association between AIP and stroke; (K) The association between LAP and hypertension; (L) The association between LAP and heart failure; (M) The association between LAP and coronary heart disease; (N) The association between LAP and angina; and (O) The association between LAP and stroke. AIP = atherogenic index of plasma, LAP = lipid accumulation product, NHHR = non-HDL-C/HDL-C ratio.

The results from RCS curves and threshold effect analyses further clarified the relationships between lipid metabolism indices and cardiovascular/cerebrovascular diseases (Fig. 2). NHHR, AIP, and LAP exhibited nonlinear associations with hypertension (*P* for nonlinearity < .05), while their associations with heart failure were linear (*P* for overall < .001, *P* for nonlinearity > .05). For coronary heart disease, NHHR and LAP showed nonlinear associations (*P* for nonlinearity < .05), whereas AIP demonstrated a linear association (*P* for overall < .001, *P* for nonlinearity > .05). In analyses of angina, LAP exhibited a nonlinear association (*P* for nonlinearity < .05), while NHHR and AIP displayed linear relationships (*P* for overall < .001, *P* for nonlinearity > .05). For stroke, NHHR and AIP demonstrated nonlinear associations (*P* for nonlinearity < .05), with no significant association observed for LAP.

Threshold effect analyses identified inflection points for all nonlinear relationships (Tables S1–S3, Supplemental Digital Content, https://links.lww.com/MD/R420). NHHR showed inflection points at 5.39 for hypertension, 5.17 for coronary heart disease, and 2.31 for stroke (Table S1, Supplemental Digital Content, https://links.lww.com/MD/R420). AIP exhibited inflection points at 0.42 for hypertension and 0.22 for stroke (Table S2, Supplemental Digital Content, https://links.lww.com/MD/R420). LAP demonstrated inflection points at 6.43 for hypertension, 15.88 for coronary heart disease, and 15.73 for angina (Table S3, Supplemental Digital Content, https://links.lww.com/MD/R420).

### 3.4. Stability assessment of NHHR, AIP, and LAP in predicting hypertension, heart failure, coronary heart disease, angina, and stroke

**Figure 3.**
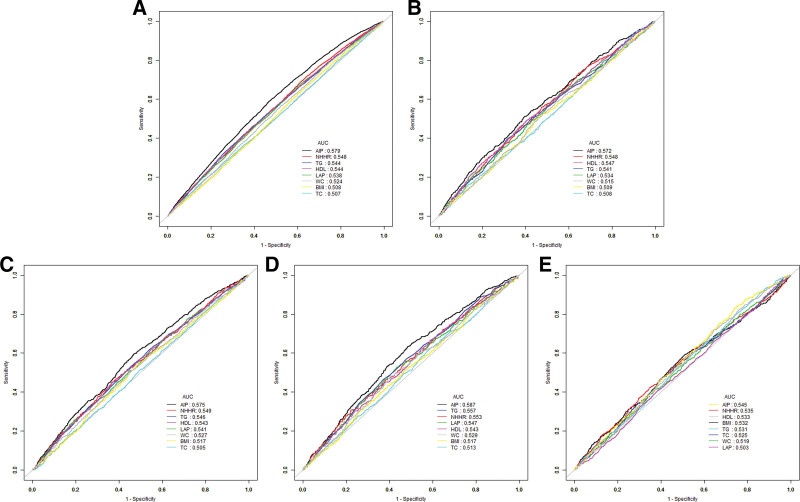
Receiver operating characteristic (ROC) curve analysis for predicting CVD. (A) ROC curve analysis for predicting hypertension; (B) ROC curve analysis for predicting heart failure; (C) ROC curve analysis for predicting coronary heart disease; (D) ROC curve analysis for predicting angina; and (E) ROC curve analysis for predicting stroke. CVD = cardiovascular disease.

To evaluate the robustness of the models, this study compared the predictive capabilities of 3 lipid metabolism indices – NHHR, AIP, and LAP – for hypertension, heart failure, coronary heart disease, angina, and stroke. The results demonstrated that AIP and NHHR exhibited superior and stable predictive performance for cardiovascular disease (CVD) risk compared to traditional lipid markers (HDL-C, TC, TG) and obesity indices (BMI, WC), as evidenced by larger area under the (receiver-operating characteristic) curve (AUC) values (AIP: AUC = 0.579, 0.572, 0.575, 0.587, 0.545; NHHR: AUC = 0.548, 0.548, 0.549, 0.553, 0.535). Additionally, LAP showed moderate predictive utility for CVDs (AUC = 0.538, 0.534, 0.541, 0.547) (Fig. 3), though its predictive capacity for stroke was notably weaker (AUC = 0.503).

### 3.5. Subgroup analyses

**Figure 4.**
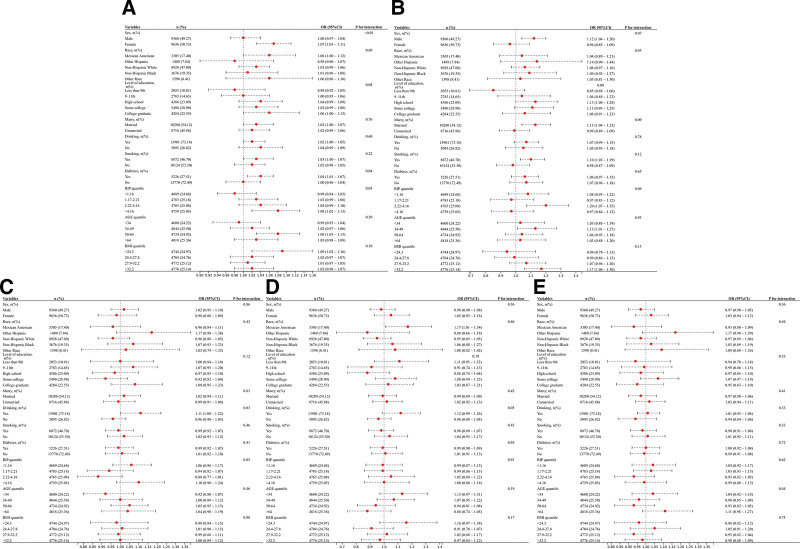
Subgroup analysis of NHHR and CVD. (A) Subgroup analysis of NHHR and hypertension; (B) Subgroup analysis of NHHR and heart failure; (C) Subgroup analysis of NHHR and coronary heart disease; (D) Subgroup analysis of NHHR and angina; and (E) Subgroup analysis of NHHR and stroke. CVD = cardiovascular disease, NHHR = non-HDL-C/HDL-C ratio.

**Figure 5.**
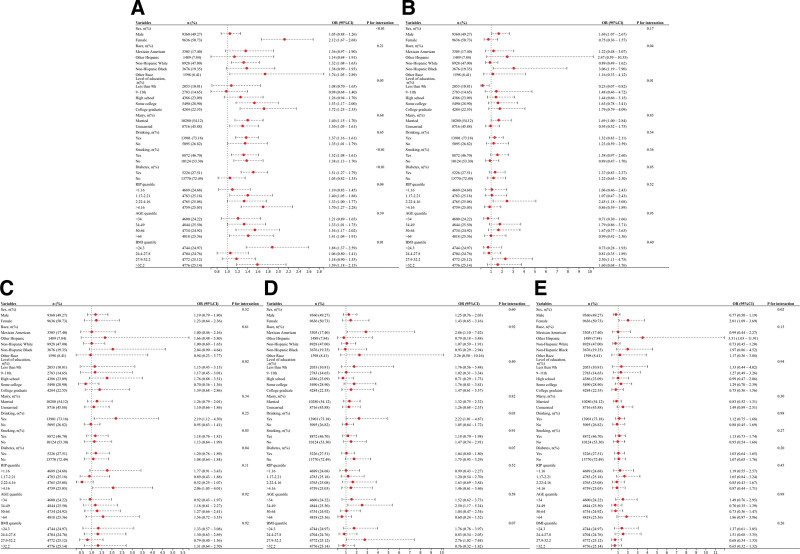
Subgroup analysis of AIP and CVD. (A) Subgroup analysis of AIP and hypertension; (B) Subgroup analysis of AIP and heart failure; (C) Subgroup analysis of AIP and coronary heart disease; (D) Subgroup analysis of AIP and angina; and (E) Subgroup analysis of AIP and stroke. AIP = atherogenic index of plasma, CVD = cardiovascular disease.

**Figure 6.**
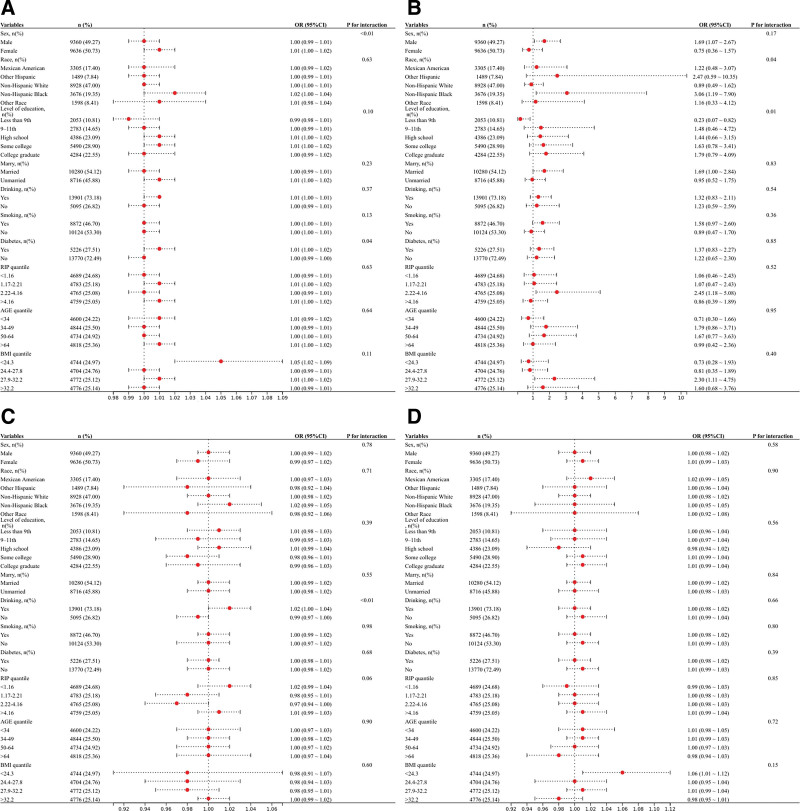
Subgroup analysis of LAP and CVD. (A) Subgroup analysis of LAP and hypertension; (B) Subgroup analysis of LAP and heart failure; (C) Subgroup analysis of LAP and coronary heart disease; (D) Subgroup analysis of LAP and angina. CVD = cardiovascular disease, LAP = lipid accumulation product.

Subgroup analyses were conducted to determine whether the associations between lipid metabolism indices and cardiovascular/cerebrovascular diseases were stable across subgroups. For hypertension, significant interactions were observed between NHHR and sex (male/female), diabetes (yes/no), and PIR (*P* < .05) (Fig. 4A). AIP demonstrated interactions with sex (male/female), education level, smoking status (yes/no), diabetes (yes/no), and BMI (*P* < .05) (Fig. 5A), while LAP exhibited significant interactions with sex (male/female), diabetes (yes/no), and BMI (*P* < .05) (Fig. 6A). In heart failure analyses, AIP and LAP showed interactions with race and education level, respectively (*P* < .05) (Figs. 5B and 6B), whereas no significant interactions were found for NHHR (*P* > .05) (Fig. 4B). For coronary heart disease, NHHR and LAP displayed interactions with alcohol consumption (yes/no) (*P* < .05) (Figs. 4C and 6C), while AIP was associated with diabetes status (yes/no) (*P* < .05) (Fig. 5C). Notably, AIP showed significant interactions with angina risk in alcohol consumers (yes/no) (*P* < .05) and sex-specific associations (male/female) with stroke risk (*P* < .05) (Fig. 5D and E). No significant interactions were observed between NHHR/LAP and angina/stroke subgroups (*P* > .05) (Fig. 4D and E and Fig. 6D).

## 4. Discussion

This study is the first to reveal the association patterns between novel lipid markers (NHHR, AIP, LAP) and cardiovascular/cerebrovascular diseases using the NHANES database. The results demonstrated nonlinear associations of NHHR, AIP, and LAP with hypertension, with inflection points at 5.39, 0.42, and 6.43, respectively, while their associations with heart failure exhibited linear increasing trends. For coronary heart disease, NHHR and LAP showed nonlinear associations, whereas AIP displayed a linear positive correlation. In stroke, only NHHR and AIP demonstrated nonlinear associations, with no significant correlation observed for LAP. Additionally, NHHR and AIP exhibited linear increases in angina risk, while LAP showed a nonlinear association with angina. These findings indicate heterogeneous predictive patterns of lipid markers for specific cardiovascular/cerebrovascular diseases. These associations remained robust after adjusting for covariates (sex, age, race, education, marital status, PIR, smoking, alcohol consumption, diabetes, and BMI). Subgroup interaction analyses further validated the stability of these associations: sex and diabetes significantly modified associations in hypertension; race and education influenced heart failure associations for AIP and LAP; alcohol consumption affected coronary heart disease associations for NHHR and LAP; diabetes status modified AIP’s association with coronary heart disease; alcohol use and sex-specific interactions were observed for angina and stroke risks, respectively. The threshold effects identified in this study, particularly the inflection points found in the RCS analysis, are of interest. For example, the NHHR inflection point for hypertension at 5.39 suggests that the relationship between NHHR and hypertension may change at this level. However, it is important to note that these inflection points do not directly indicate a clinical intervention threshold. Instead, they represent points where the relationship between lipid metabolism markers and disease outcomes may exhibit significant changes. Further studies are needed to explore whether these inflection points correspond to actual clinical intervention thresholds, and whether they can be used to guide treatment decisions. Predictive stability tests revealed that AIP exhibited the most stable and reliable CCVDs risk prediction trend, while NHHR and LAP also demonstrated moderate predictive stability. The identification of these threshold effects in lipid metabolism markers suggests that clinical guidelines might need to incorporate specific cutoff values to improve risk stratification in cardiovascular disease prevention. However, further prospective studies are necessary to validate these thresholds and their role in clinical decision-making. These results support and extend the study hypothesis, confirming correlations between novel lipid markers and cardiovascular/cerebrovascular disease risks, with potential utility in predicting disease onset. This underscores the importance of early intervention targeting lipid metabolism abnormalities in cardiovascular health management.

Recent studies have increasingly focused on the relationship between cardiovascular/cerebrovascular diseases and lipid metabolism.^[23,24]^ In this study, significant correlations were observed between cardiovascular/cerebrovascular diseases and novel lipid markers (NHHR, AIP, and LAP), which could be predicted using these markers. These associations align with findings from prior research. The American Heart Association’s expert consensus on cardiovascular events identifies non-HDL-C as both a target value and an independent predictor for high-risk populations. NHHR, a novel lipid marker reflecting the ratio of non-HDL-C to HDL-C, captures the interplay between atherogenic and protective lipid components.^[25,26]^ Non-HDL-C includes chylomicrons, very-LDL-C remnants, intermediate-density lipoprotein cholesterol, LDL-C and lipoprotein(a). very-LDL-C and LDL-C are taken up via receptors on vascular endothelial cells, leading to cholesterol accumulation in the arterial wall. Ox-LDL, derived from LDL-C within the arterial wall, promotes endothelial apoptosis, impairs vasodilation, triggers pro-inflammatory cytokine release, disrupts endothelial function, and increases thrombosis and plaque rupture risks.^[27,28]^ Structural vascular changes from these processes elevate vascular resistance, contributing to hypertension. AIP, which integrates HDL-C and TG, demonstrates greater stability compared to individual lipid measures. Previous studies indicate that AIP outperforms conventional lipid markers in predicting stroke risk,^[29]^ likely due to TRL modifications during dyslipidemia. TRLs infiltrate the vascular wall, exacerbate foam cell formation and plaque development, stimulate monocyte-mediated inflammatory cell recruitment, and destabilize plaques, thereby accelerating cardiovascular/cerebrovascular events.^[30]^

In this study, positive correlations between lipid indices (NHHR, AIP, LAP) and hypertension, heart failure, coronary heart disease, angina, and stroke persisted even after full adjustment for covariates, confirming their stability. Subgroup interaction tests further validated these associations across groups while revealing heterogeneity in specific subgroups. For instance, sex differences in hypertension correlations may stem from sex-specific endocrine factors. Estrogen regulates vascular function through vasodilation promotion, vascular remodeling inhibition, and modulation of the renin-angiotensin-aldosterone system and sympathetic nervous activity, exerting protective effects on blood pressure regulation in premenopausal women.^[31,32]^ Abnormal glucose levels activate vascular injury signaling and the polyol pathway, impairing vascular function and elevating blood pressure.^[33]^ Epidemiological data show that African Americans have a 1.75-fold higher heart failure incidence and earlier onset than Whites, potentially linked to genetic susceptibility to salt-sensitive hypertension variants that predispose to cardiac structural and functional abnormalities.^[34]^ Lower education levels are associated with inadequate recognition of early heart failure symptoms and discontinuation of beta-blockers and angiotensin-receptor neprilysin inhibitor medications, indirectly increasing heart failure risk,^[35]^ which explains the significant interactions observed for race and education level in heart failure prevalence. Hyperglycemia promotes coronary heart disease via multiple mechanisms: enhanced foam cell formation, advanced glycation end product accumulation, endothelial damage, and accelerated atherosclerosis.^[36]^ Advanced glycation end products bind to vascular collagen and elastin, increasing arterial stiffness and disrupting vascular structure/function.^[37]^ Hyperglycemia also induces hypercoagulability and microcirculatory dysfunction, altering hemodynamics and raising cardiovascular event risks.^[38]^ Excessive alcohol consumption elevates coronary heart disease risk through ethanol-induced myocardial oxidative damage and alpha-adrenergic receptor-mediated vasospasm, contributing to angina.^[39,40]^ Subgroup analyses for stroke revealed sex-specific associations, with postmenopausal women accounting for 60% of stroke cases, likely due to estrogen depletion accelerating LDL oxidation and plaque ulceration,^[41,42]^ compounded by higher autoimmune disease prevalence (e.g., lupus) and hypercoagulability in women.^[43,44]^

In summary, the relationships between novel lipid markers (NHHR, AIP, LAP) and cardiovascular/cerebrovascular diseases result from multifactorial interactions and demonstrate stable associations. While further mechanistic studies are needed, this research provides insights into lipid metabolic factors underlying cardiovascular/cerebrovascular diseases, supporting early prevention and treatment strategies for at-risk populations.

## 5. Strengths and limitations

This cross-sectional study addresses gaps in traditional cardiovascular risk prediction by exploring novel lipid markers (NHHR, AIP, LAP) using NHANES data. It provides the first evidence of their stable associations with cardiovascular/cerebrovascular diseases, validated through multivariable adjustments and subgroup analyses. However, limitations include potential non-differential misclassification bias from single lipid measurements and residual confounding by unmeasured factors (e.g., gut microbiota, epigenetics). Causality cannot be established due to the cross-sectional design. Future large-scale cohort studies and mechanistic investigations are needed to elucidate causal pathways and molecular interactions between lipid metabolism and cardiovascular/cerebrovascular diseases.

## 6. Conclusion

This study found that NHHR and AIP were significantly associated with the risk of CCVDs, while LAP was associated with cardiovascular risks but showed no significant correlation with stroke. The risk of CCVDs increased progressively with elevated levels of these lipid metabolism markers. Future prospective studies should employ large-scale cohort research to elucidate causal relationships and underlying molecular mechanisms, and evaluate targeted intervention strategies based on these lipid metabolism indices, including designing clinical trials on statin dose adjustment, to advance the clinical application of personalized lipid management strategies.

## Author contributions

**Conceptualization:** Tonglu Su, Shilong Xin, Yu Zhang.

**Data curation:** Benyin Wang, Xiaojuan Wang, Yingli Zhang.

**Writing – original draft:** Limei Guan.

**Writing – review & editing:** Tao Sun.

## Supplementary Material


