# Supplementary material for: Association of novel lipid markers with cardiovascular and cerebrovascular disease risk: A cross-sectional NHANES 1999 to 2018 study
Source: Medicine (Baltimore). 2026 Feb 28;105(9):e45025. doi: 10.1097/MD.0000000000045025 (PMC12956176; doi:10.1097/MD.0000000000045025)
Supplement: Supplementary file 1 [file medi-105-e45025-s001.docx]

**Table S1. Threshold effect analysis of NHHR on CCVDs.**

| NHHR | | | | | |
| --- | --- | --- | --- | --- | --- |
| Hypertension | | Coronary heart disease | | Stroke | |
| A straight-line effect | 1.09 (1.07, 1.11) <0.0001 | A straight-line effect | 1.05 (1.00, 1.10) 0.0390 | A straight-line effect | 1.02 (0.96, 1.07) 0.5513 |
| Fold points (K) | 5.39 | Fold points (K) | 5.17 | Fold points (K) | 2.31 |
| < K-segment effect 1 | 1.13 (1.10, 1.16) <0.0001 | < K-segment effect 1 | 1.14 (1.07, 1.22) 0.0002 | < K-segment effect 1 | 1.49 (1.15, 1.94) 0.0028 |
| < K-segment effect 2 | 0.96 (0.91, 1.02) 0.2218 | < K-segment effect 2 | 0.78 (0.61, 0.98) 0.0369 | < K-segment effect 2 | 0.95 (0.88, 1.03) 0.2095 |
| Log likelihood ratio tests | <0.001 | Log likelihood ratio tests | <0.001 | Log likelihood ratio tests | 0.002 |

**Table S2. Threshold effect analysis of AIP on CCVDs.**

| AIP | | | |
| --- | --- | --- | --- |
| Hypertension |  | Stroke |  |
| A straight-line effect | 2.13 (1.94, 2.33) <0.0001 | A straight-line effect | 1.33 (1.06, 1.68) 0.0155 |
| Fold points (K) | 0.42 | Fold points (K) | 0.22 |
| < K-segment effect 1 | 3.24 (2.75, 3.82) <0.0001 | < K-segment effect 1 | 4.02 (1.91, 8.45) 0.0002 |
| < K-segment effect 2 | 1.32 (1.10, 1.57) 0.0024 | < K-segment effect 2 | 0.90 (0.64, 1.28) 0.5686 |
| Log likelihood ratio tests | <0.001 | Log likelihood ratio tests | 0.001 |

**Table S3. Threshold effect analysis of LAP on CCVDs.**

| LAP | | | | | |
| --- | --- | --- | --- | --- | --- |
| Hypertension | | Coronary heart disease | | Angina | |
| A straight-line effect | 1.14 (1.08, 1.20) <0.0001 | A straight-line effect | 1.12 (1.01, 1.23) 0.0298 | A straight-line effect | 1.10 (1.00, 1.20) 0.0446 |
| Fold points (K) | 6.43 | Fold points (K) | 15.88 | Fold points (K) | 15.73 |
| < K-segment effect 1 | 2.34 (1.92, 2.85) <0.0001 | < K-segment effect 1 | 1.55 (1.29, 1.87) <0.0001 | < K-segment effect 1 | 1.56 (1.26, 1.95) <0.0001 |
| < K-segment effect 2 | 1.04 (0.99, 1.10) 0.1294 | < K-segment effect 2 | 0.70 (0.48, 1.04) 0.0746 | < K-segment effect 2 | 0.77 (0.53, 1.12) 0.1707 |
| Log likelihood ratio tests | <0.001 | Log likelihood ratio tests | <0.001 | Log likelihood ratio tests | <0.001 |
